# Differences in Dietary Composition and Interspecific Competition Among Large Carnivores on the Qinghai–Xizang Plateau

**DOI:** 10.1002/ece3.73037

**Published:** 2026-02-02

**Authors:** Dong Wang, Quanbang Li, Xinming Lian

**Affiliations:** ^1^ Key Laboratory of Adaptation and Evolution of Plateau Biota, Northwest Institute of Plateau Biology, Chinese Academy of Sciences Xining China; ^2^ School of Geographical Science Qinghai Normal University Xining China; ^3^ University of Chinese Academy of Sciences Beijing China; ^4^ Qinghai Provincial Key Laboratory of Animal Ecological Genomics Xining China

**Keywords:** DNA metabarcoding, interspecific competition, large carnivores, Qinghai–Xizang Plateau, trophic niche

## Abstract

Understanding and quantifying the dietary composition of large carnivores is crucial for elucidating their functional roles within ecosystems including their top–down regulation of prey populations and their interactions with sympatric carnivore species. In this study, we employed DNA metabarcoding to analyze the vertebrate components of the diets of three sympatric large carnivores, snow leopard *Panthera uncia* , wolf *Canis lupus* , and brown bear *Ursus arctos* , with particular emphasis on assessing potential interspecific competition in the Sanjiangyuan Region (SR) of the Qinghai–Xizang Plateau. Analysis revealed 11, 16, and 17 prey species in the diets of wolves, snow leopards, and brown bears, respectively. Domestic yak ( *Bos grunniens* ) was the most frequently detected prey item in the diets of both wolves (Relative Read Abundance; *RRA* = 52.29%) and snow leopards (*RRA* = 25.42%), whereas brown bears primarily consumed plateau pikas ( *Ochotona curzoniae* ; *RRA* = 43.10%) and Himalayan marmots ( *Marmota himalayana* ; RRA = 19.88%). Although high dietary niche breadth overlap was observed between snow leopards and wolves (*O*_*jk*_ = 0.76) and between snow leopards and brown bears (*O*_*jk*_ = 0.79), potential interspecific competition may be mitigated through differential prey selection and varying consumption intensities. The relatively low dietary overlap between wolves and brown bears (*O*_*jk*_ = 0.32) implies that these two species likely coexist by partitioning trophic resources. Moreover, the substantial proportion of livestock found in the diet of these large carnivores indicates potential presence of significant human–carnivore conflict in the SR. Combined with previous findings, our results support the hypothesis that the abundance and size‐class availability of ungulate prey are key factors enabling the sympatric existence of these three apex predators.

## Graphical abstract

This study elucidated the mechanisms underlying regional coexistence among the snow leopard ( *Panthera uncia* ), wolf ( *Canis lupus* ), and brown bear ( *Ursus arctos* ) through the analysis of their trophic niches. The findings provide a scientific foundation for further investigation into the mechanisms that maintain carnivore communities in high‐altitude ecosystems.

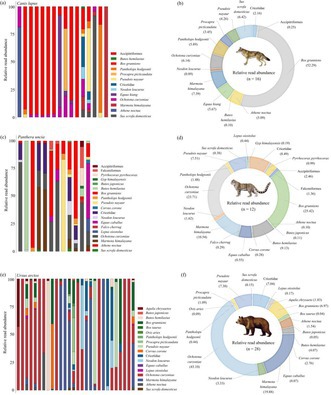

## 1 Introduction

The fundamental and direct interactions among the various components of an ecosystem are primarily mediated and realized through trophic relationships between species (Prigioni et al. 2008). Food serves as a critical link between animals and their environment, providing not only the nutrients and energy necessary for survival and reproduction but also forming the basis for the establishment of various interspecific relationships within animal communities (Mysterud 2000). According to optimal foraging theory, foraging behavior represents a trade‐off between the energetic costs associated with activities such as searching for, pursuing, and capturing prey, and the energetic benefits derived from food consumption (Perry and Pianka 1997). Large carnivores typically prioritize prey items that offer high energetic returns and are readily accessible (Lu et al. 2023). Consequently, when populations of wild prey decline or when livestock become more easily available, large carnivores are more likely to shift their predation preference toward domestic animals, resulting in increased reliance on livestock.

As apex predators occupying the highest trophic level in food chains, large carnivores exert top‐down regulatory effects on prey populations through trophic cascades (Ripple et al. 2014). This ecological regulation maintains herbivore populations at relatively stable levels, thereby promoting species interactions and preserving the functional integrity of ecological systems (Roemer et al. 2009). The composition and structure of large carnivore communities are shaped by both direct and indirect interactions among species within the same guild (Linnell and Strand 2000). Compared to other vertebrate groups, large carnivores have significantly higher energetic requirements (Gomez‐Ortiz et al. 2015). These demands intensify interspecific competition among sympatric carnivore species with overlapping dietary preferences, often manifesting as both exploitative and interference competition (Karanth et al. 2017; Rodríguez‐Luna et al. 2025). Intraguild competition may constrain certain species to suboptimal niches, potentially compromising their fitness and survival (Du Preez et al. 2017). The abundance of prey resources within an ecosystem plays a critical role in determining carnivore guild density. Furthermore, selective predation can promote coexistence among sympatric carnivores by enhancing ecological segregation (Harihar et al. 2011).

Diet analysis provides valuable insights into key aspects of animal ecology and evolution, and supports the development of effective conservation strategies (de Sousa et al. 2019). For large carnivores, prey availability represents their most fundamental ecological requirement and a primary determinant of population size and spatial distribution (Stepkovitch et al. 2022). Niche breadth refers to the variety of food resources utilized by a species and is considered an important metric for assessing dietary specialization (Segurado et al. 2011; Vissia et al. 2023). Species with narrow niche breadths are regarded as relatively specialized, whereas those with broader niche breadths are classified as generalist species (Smith et al. 2011). Research has demonstrated that seasonal variation significantly influences the trophic niche breadth of animals. During periods of food abundance, the trophic niche breadth typically decreases, while in seasons marked by limited prey availability, it tends to increase (MacArthur and Pianka 1966). Furthermore, when prey availability is high, exploitative competition among carnivores is reduced. Under such conditions, carnivores preferentially target large ungulates that provide higher energy returns, resulting in narrower trophic niche breadths and greater dietary overlap (Kumaraguru et al. 2011; Wang et al. 2014). This pattern aligns with the predictions of the resource abundance hypothesis, which posits that when large prey resources are abundant, sympatric carnivores are more likely to concentrate their foraging efforts on these preferred resources, thereby increasing dietary overlap (Lanszki et al. 2019). Conversely, during periods of prey scarcity, exploitative competition intensifies, prompting carnivores to shift their diets toward smaller prey species with lower energy yields. Such dietary adjustments lead to broader trophic niche breadths and reduced dietary overlap (Li and Wang 2022; Steinmetz et al. 2021). However, among sympatric specialist carnivores, dietary overlap may increase under food‐limited conditions, as species are constrained to exploit the same limited pool of prey resources.

The Qinghai–Xizang Plateau exhibits pronounced elevational gradients that have contributed to the development of unique biodiversity, making this region one of the global hotspots with the highest densities of carnivore species (Pimm et al. 2014). The Sanjiangyuan Region (SR), located in the heart of the Plateau, contains vast and contiguous areas of high‐quality habitat suitable for large carnivores such as snow leopards ( *Panthera uncia* ), wolves ( *Canis lupus* ), and brown bears ( *Ursus arctos* ) (Dai et al. 2019; Li et al. 2013). These three apex predators are broadly distributed and coexist sympatrically across the region. Recent studies have indicated that snow leopards and wolves primarily rely on the predation of large ungulates, whereas the brown bears, being opportunistic omnivores, maintain a highly diverse and complex dietary composition (Lu et al. 2023). Nevertheless, there remains a notable paucity of research examining the interspecific interactions among these large carnivores in the SR from the perspective of dietary differentiation.

Due to the limited availability of environmental resources, sympatric species have developed distinct foraging strategies over evolutionary timescales to fulfill their fundamental needs (Andheria et al. 2007; Wang et al. 2014). Food resources represent a critical factor in interspecific competition and play a central role in shaping the mechanisms that facilitate coexistence within those ecological communities. In this study, we analyzed the dietary composition, trophic niche breadth, and levels of dietary overlap among sympatric snow leopards, wolves, and brown bears in the SR. Our findings offer a theoretical basis for understanding interspecific interactions and guiding the development of effective conservation strategies for large carnivores on the Qinghai–Xizang Plateau. This study examined the following predictions: (1) among the three large carnivores, livestock constitute the highest proportion of the diet in wolves, followed by snow leopards, whereas brown bears exhibit the lowest proportion (Dai et al. 2020; Wang et al. 2024); and (2) as an omnivorous and opportunistic forager, the brown bear exhibits minimal dietary overlap with both the snow leopard and the wolf, while the latter two species demonstrate substantial dietary overlap.

## 2 Materials and Methods

### 2.1 Study Area

**FIGURE 1:**
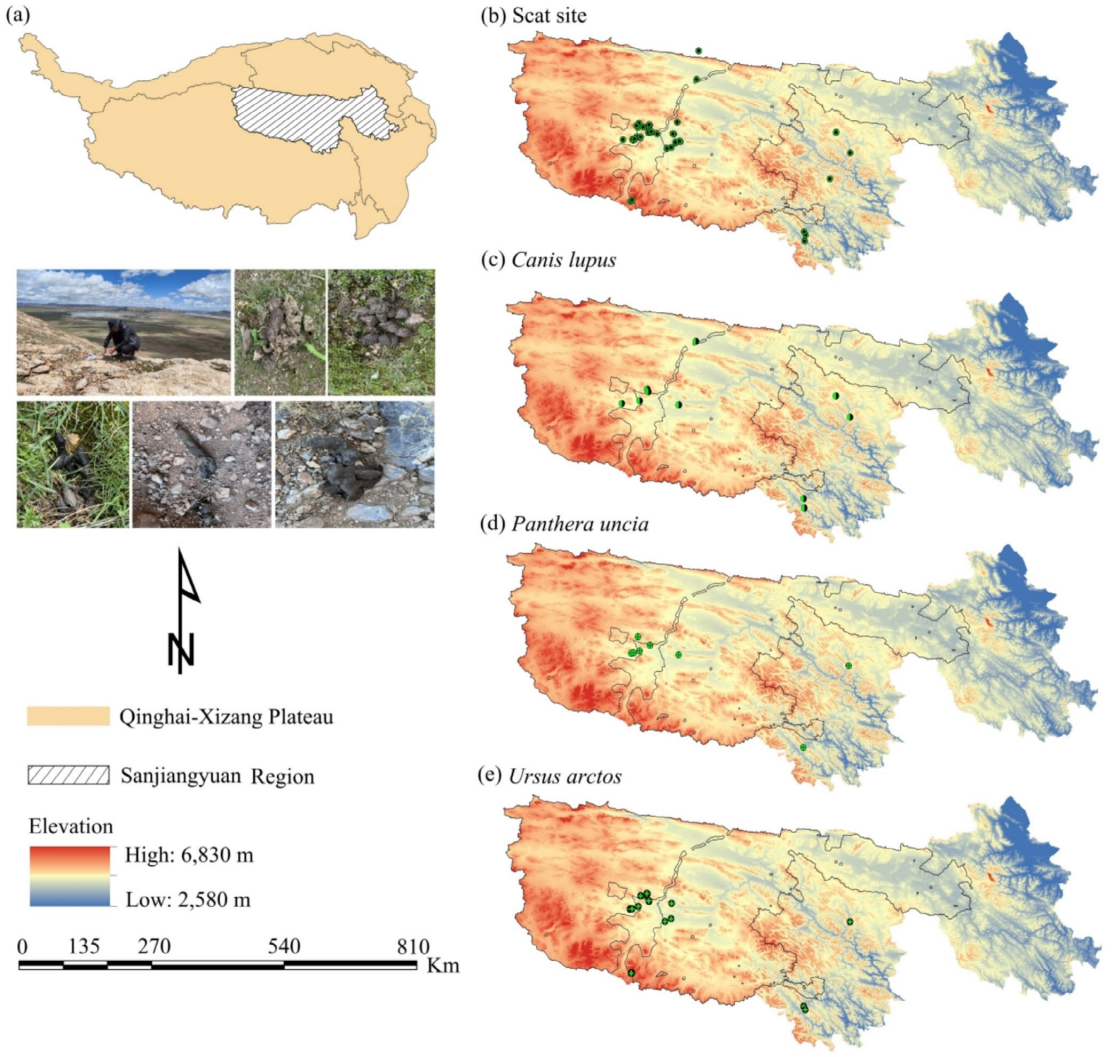
Locations of collected scats from large carnivores in the Sanjiangyuan Region.

The SR (31°39′~36°16′ N, 89°24′~102°23′ E; Figure 1) is situated in the southern part of Qinghai Province, China, and lies within the hinterland of the Qinghai–Xizang Plateau (Lu et al. 2025). It serves as the headwater region of the Yangtze, Yellow, and Lancang Rivers and functions as a critical area for carbon sequestration, water conservation, and ecological security in China (Song et al. 2022). The SR is characterized by vast, rugged, and topographically complex landscapes. Overall, elevation increases from the southeast to the northwest, with an average altitude of approximately 4400 m above sea level. The region experiences a typical plateau continental climate and constitutes an essential component of the Qinghai–Xizang Plateau's climate system. It has an annual mean temperature ranging from −5.7°C to 7.8°C, with annual precipitation varying between 262.2 mm and 772.8 mm, exhibiting a spatial gradient of increasing rainfall from the northwest toward the southeast (Dai et al. 2022). Since the 1960s, the SR has experienced a significant warming and humidification trend (Liang et al. 2013).

The mammalian fauna in the SR is distinguished by a high degree of species diversity, encompassing numerous endemic species, key nationally protected species, and species classified as threatened (Xi et al. 2020). The SR features distinctive topographical characteristics and a wide range of habitat types, making it a core distribution area for large carnivores such as snow leopards, wolves, and brown bears. Additionally, the region supports a sizeable population of wild ungulates, including bharal ( *Pseudois nayaur* ), kiang ( *Equus kiang* ), Tibetan gazelle ( *Procapra picticaudata* ), and Tibetan antelope ( *Pantholops hodgsonii* ), which constitute a significant prey base for these apex carnivores.

### 2.2 Fecal Sample Collection

Given that wolves, snow leopards, and brown bears primarily inhabit high‐altitude plateau mountainous regions above 3000 m in the SR, which are characterized by rugged terrain and extremely limited road access, and given their elusive behavior and low population densities, direct observation of predatory behaviors is often insufficient for obtaining reliable dietary data. Scat collection along wildlife trails, saddles, and rocky outcrops has proven to be an effective method for monitoring the diet and population dynamics of large carnivores (Janecka et al. 2011). Prior to collecting carnivore fecal samples along line transects, we conducted interviews with local herdsmen or ecological rangers familiar with the distribution of local wildlife and the terrain characteristics. On the basis of this information and the local topography, we established survey transects that ranged from 3 to 5 km in length, with adjacent transects spaced approximately 1 km apart. During the field survey, we systematically walked along each transect and collected all fecal samples suspected to originate from large carnivores within visible range on both sides. The number of transects surveyed per day, ranging from one to two, was determined by weather conditions and the route difficulty. In December 2023 and July 2024, we collected putative fecal samples from snow leopards, wolves, and brown bears in the SR using the line transect method. To maximize the representativeness and genetic diversity of sampled individuals, field surveys were conducted along ridge lines and well‐established animal trails. When multiple morphologically similar fecal samples were encountered within a 1 km radius, only one sample was collected to reduce the probability of sampling duplicates from the same individual.

To ensure the success of molecular analysis, relatively fresh fecal samples exhibiting moist surfaces and darker pigmentation were selected. During sample handling, disposable gloves were worn to ensure contamination control. Three subsamples were collected from distinct regions of the fecal sample and transferred into a 50 mL sterile tube, filling approximately three–quarters of the tube. The tube was then labeled with the sampling date and sample ID. In addition, detailed field information, including GPS coordinates, altitude, and adjacent landscape features, was recorded on standardized sampling forms. Immediately after collection, samples were submerged in absolute ethanol and fixed for a period of 24 h, following which the ethanol was carefully removed. Silica gel desiccant was subsequently added to promote complete desiccation. Upon completion of the field preservation process, all samples were transported to the laboratory and stored at −20°C for subsequent analysis.

### 2.3 Species Identification and Dietary Analysis

In this study, metabarcoding was employed to identify host species and dietary composition. Genomic DNA was extracted from fecal samples using the QIAamp Fast DNA Stool Mini Kit (QIAGEN, Hilden, Germany), following the manufacturer's instructions with strict adherence. During the fecal DNA extraction process, CTAB and starch were employed to remove PCR inhibitors. Subsequently, DNA was purified through multiple phenol‐chloroform extractions. Subsequently, DNA concentration was measured using a Qubit fluorometer to ensure accurate quantification. The 12S V5‐F/R primers pair (F_12SV5: 5′‐TAGAACAGGCTCCTCTAG‐3′; R_12SV5: 5′‐TTAGATACCCCACTATGC‐3′) was utilized for amplification to determine both host species identity and dietary components from the samples (Hacker et al. 2021; Wang et al. 2025). This universal vertebrate primer is widely used in dietary studies of various carnivores due to its broad taxonomic applicability and high resolution. Amplification of the mitochondrial 12S RNA V5 region from fecal DNA enables precise characterization of the dietary composition of large carnivores (Cong et al. 2024; Xiong et al. 2017).

Due to the relatively low abundance of food‐derived DNA in fecal samples, combined with challenges such as DNA degradation and the presence of PCR inhibitors, shorter amplification products are preferred to improve the success rate of dietary DNA amplification (Lu et al. 2019). In this study, blocking primers were not used during the dietary DNA amplification process to suppress predator DNA amplification. To minimize the influence of the host DNA on dietary analysis, sequencing depth was increased to 300,000 reads per sample. Furthermore, after confirming the host species of each fecal sample, host‐specific sequences were removed prior to downstream analyses.

PCR amplification was carried out in a total reaction volume of 25 μL, consisting of 6 μL of DNA template (10–100 ng/μL), 15 μL of 2 × EasyTaq PCR SuperMix, 0.2 μM of the 12S V5–F primer, and 0.2 μM of the 12S V5–R primer. The thermal cycling profile was as follows: an initial denaturation at 95°C for 5 min; 35 cycles of denaturation at 95°C for 30 s, annealing at 60°C for 30 s, and extension at 72°C for 30 s; followed by a final extension at 72°C for 10 min. In each PCR batch, both a fecal DNA extraction blank and a no‐template PCR control were included to monitor potential DNA contamination.

Library preparation was conducted in accordance with the standardized protocol provided by the ALFA‐SEQ Amplicon Library Prep Kit. The quality and integrity of the constructed libraries were assessed using both the Qubit fluorimeter and the QSEP400 high‐throughput nucleic acid/protein analysis system to ensure sequencing readiness. Thereafter, paired‐end sequencing was performed on the Illumina Nova‐Seq 6000 platform, with a read length of 250 base pairs (bp) for each end. All library preparation and sequencing procedures were outsourced to Guangdong Magigene Biotechnology Co. Ltd. (Guangzhou, China), a certified service provider specializing in high‐throughput sequencing.

The raw reads generated from second‐generation sequencing were initially processed through quality control using the Fastp software (version 0.12.4). This procedure included adapter trimming and the elimination of low‐quality sequences, which were defined according to the following criteria: (1) reads with a length shorter than 100 bp; (2) reads containing more than five ambiguous bases (N); (3) reads with an average base quality score below 20; and (4) reads in which the proportion of low‐quality bases exceeded 3% (Chen et al. 2018). The resulting high‐quality paired‐end reads were then assembled using USEARCH software (version 11.0.667), with a default subsampling size of 350,000 reads; if fewer reads were available, all reads were included in the analysis (Edgar 2010). Following assembly, VSEARCH software (version 2.8.1) was applied to remove primer sequences based on end alignment, as well as sequences exhibiting a sequencing error rate greater than 1% (Torbjørn et al. 2016).

Chimeric sequences within the representative sequences were eliminated using the uchime3_denovo algorithm implemented into USEARCH software. Operational Taxonomic Unit (OTU) clustering was conducted using the OTU algorithm to generate representative OTU sequences. Subsequently, the usearch_global algorithm was applied to align the assembled sequences from all merged samples against the representative OTU sequence set, requiring minimum sequence coverage of 97% and a similarity threshold of 99%, thereby producing the final OTU table. The obtained OTU sequences were then subjected to BLAST analysis (version 2.2.31) against reference sequences in the target gene metabarcoding database (NCBI database, https://blast.ncbi.nlm.nih.gov/Blast.cgi). Taxonomic assignments at the family, genus, and species levels were made based on a defined similarity threshold. Sequences with an alignment length below 90% or a similarity score lower than 80% were excluded from further analysis (Yukuto et al. 2018). As an additional verification step, the geographic distributions of the identified host and prey species were cross‐referenced with data from the IUCN Red List (https://www.iucnredlist.org/) to confirm that their ranges overlapped with the study site (Cong et al. 2024).

In the dietary composition analysis, due to significant variations in sequencing depth among different samples, it is essential to convert prey sequence counts into occurrence frequencies. Rare sequences with relative abundances below 1% in each sample were filtered out to minimize the inclusion of background sequencing artifacts. This standardization process resulted in a finalized dietary OTU table for target taxa (Mcinnes et al. 2017). A database search and sequence alignment were performed for all species identified from the samples. To reduce potential misidentification errors, we cross‐referenced the distribution catalog of bird and mammal species in the SR (Cai et al. 2019), ensuring that all reported species are indeed locally present.

### 2.4 Data Processing and Analysis

#### 2.4.1 Hill Number‐Based Diversity Analysis

In diet analysis based on DNA metabarcoding, low‐abundance sequences are commonly regarded as exogenous contaminants or false positives arising from OTU clustering. However, Hill numbers provide a unified framework for multiple diversity indices by integrating species richness and relative abundance into a single class of metrics, thereby capturing varying degrees of emphasis on richness and evenness (Alberdi and Gilbert 2019). Compared to traditional diversity indices, Hill number diversity offers consistent interpretability across different measurement scales and units. Moreover, its sensitivity to both low and high‐abundance sequences can be modulated through the order parameter *q*. As such, the application of Hill numbers in dietary analysis provides a more statistically robust approach, enabling clearer interpretation and meaningful comparison of results across studies. The formula for calculating Hill number diversity is as follows:

Dq=∑i=1spiq11−qq≠1

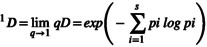

In the equation above, *s* represents the total number of species, *i* denotes the *i*th species, and *p*_*i*_ denotes its relative abundance. When *q* = 0, ^*0*^ *D* corresponds to species richness, representing the total count of species irrespective of their abundance. When *q* = 1, the formula is undefined; however, its limit as *q* approaches 1 equals the exponential of the Shannon diversity index, which emphasizes more abundant species. When *q* = 2, ^*2*^ *D* corresponds to the inverse of the Simpson diversity index, placing greater emphasis on dominant species. Hill numbers for *q* = 0, 1, and 2 were calculated for each sample using the hilldiv package (https://www.rdocumentation.org/packages/hilldiv). Furthermore, based on the prey composition OTU data for the three large carnivore species, we calculated Hill numbers across the grouped samples using the iNEXT package (https://github.com/JohnsonHsieh/iNEXT). Relationships between sample size and species richness, Shannon diversity, and Simpson diversity were subsequently assessed by generating sample‐size‐based rarefaction and extrapolation curves with 95% confidence intervals.

#### 2.4.2 Relative Abundance and Frequency Analysis of Prey

Due to the limitations in DNA barcoding resolution and database coverage, accurate species‐level identification based on individual sequence is often unattainable. Consequently, the concept of molecular operational taxonomic unit (MOTU) is employed to delineate distinct food groups, facilitating the analysis of prey composition, diversity, and intergroup variation in carnivore fecal samples.

(1) This study employed the relative read abundance (*RRA*), expressed as a percentage, to quantify the weighted mean proportion of sequence abundance for each prey taxon in fecal samples (Shao et al. 2021). The calculation formula is present as follows:

$${\mathrm{RRA}}_{i}=\frac{1}{S}\sum_{k=1}^{s}\frac{n_{i,k}}{\sum_{i=1}^{T}n_{i,k}}\times 100\%$$

Here, *S* represents the total number of fecal samples collected from the target species, *T* denotes the total number of prey occurrences, and *n*_*i,k*_ signifies the number of sequences corresponding to prey species *i* in fecal sample *k*. The sum of RRA values across all prey species within each fecal sample of the target species is normalized to 100%.

(2) We employed the frequency of occurrence (*FO*) for each prey category detected in fecal samples to characterize the relative proportion and ecological significance of specific prey types within the diet of the target species (Deagle et al. 2019; Kasper et al. 2016). The calculation formula is presented as follows:

$${\mathrm{FO}}_{i}=\frac{1}{S}\sum_{k=1}^{s}I_{i,k}\times 100\%$$

Here, *S* represents the total number of fecal samples from the target species, and *I*_*i,k*_ denotes the occurrence status of prey species *i* in fecal sample *k* (where *I*_*i,k*_ equals 1 if prey species *i* is present in fecal sample *k*, and 0 otherwise, with values accumulated accordingly). As FO indicates the frequency with which prey species *i* occurs across all samples, and since the vast majority of fecal samples contain more than two prey species, the sum of FO values does not necessarily equal 100%.

#### 2.4.3 Analysis of Dietary Differences Among Species

In this study, the ggplot2 package (https://github.com/tidyverse/ggplot2) and the ggalluvial package (https://www.rdocumentation.org/packages/ggalluvial) in R were employed to construct Sankey diagrams illustrating the dietary composition of three large carnivore species. These visualizations provide a graphical representation of the carnivores' diet profiles and the proportional abundance of various prey categories.

To further investigate interspecific dietary variation among large carnivores, this study applied the Levins index (*B*) (Hurlbert 1978) and the Pianka overlap index (*O*_*jk*_) (Peng et al. 2023; Pianka 1973) to assess trophic niche breadth and the extent of dietary overlap between species pairs. These analyses were based on the relative abundance of prey items across multiple taxonomic levels. The formulas for calculating standardized niche breadth (*B*_A_) and interspecific dietary overlap (*O*_*jk*_) are as follows:

$$B=\frac{1}{\sum_{i=1}^{n}P_{i}^{2}}$$

$$B_{A}=\left(B-1\right)/\left(n-1\right)$$

In the above formulas, parameter *B* denotes niche breadth, while *P*_*i*_ signifies the proportion of food item *i*. *B*_A_ denotes the standardized niche breadth, which ranges from 0 to 1, and n represents the total number of resource taxa (Lanszki et al. 2019).

$$O_{\mathrm{jk}}=\frac{\Sigma \;P_{\mathrm{ij}}\cdot P_{\mathrm{ik}}}{\sqrt{\Sigma \;{P_{\mathrm{ij}}}^{2}\cdot \Sigma \;{P_{\mathrm{ik}}}^{2}}}$$

In the aforementioned formula, *P*_*ij*_ and *P*_*ik*_ indicate the relative abundance of food item *i* in the diets of species *j* and *k*, respectively. The *O*_*jk*_ values range from 0 to 1. According to the evaluation criteria, an *O*_*jk*_ value of 0 indicates no dietary overlap, while an *O*_*jk*_ value of 1 indicates complete dietary overlap. An *O*_*jk*_ value exceeding 0.3 is considered to indicate significant dietary overlap, and a value greater than 0.6 is regarded as reflecting pronounced dietary overlap (Wang et al. 2025).

## 3 Results

This study utilized DNA metabarcoding to analyze a total of 160 collected fecal samples. Of these, 151 samples were successfully sequenced, generating 23,423,385 high‐quality sequences, with an average of 155,122 ± 13,703 sequences per sample. Among the target carnivore species, successful sequencing results were obtained for 16 samples from wolves, 12 samples from snow leopards, and 28 samples from brown bears, respectively.

### 3.1 Evaluation of Hill Number Diversity in Dietary Composition

**FIGURE 2:**
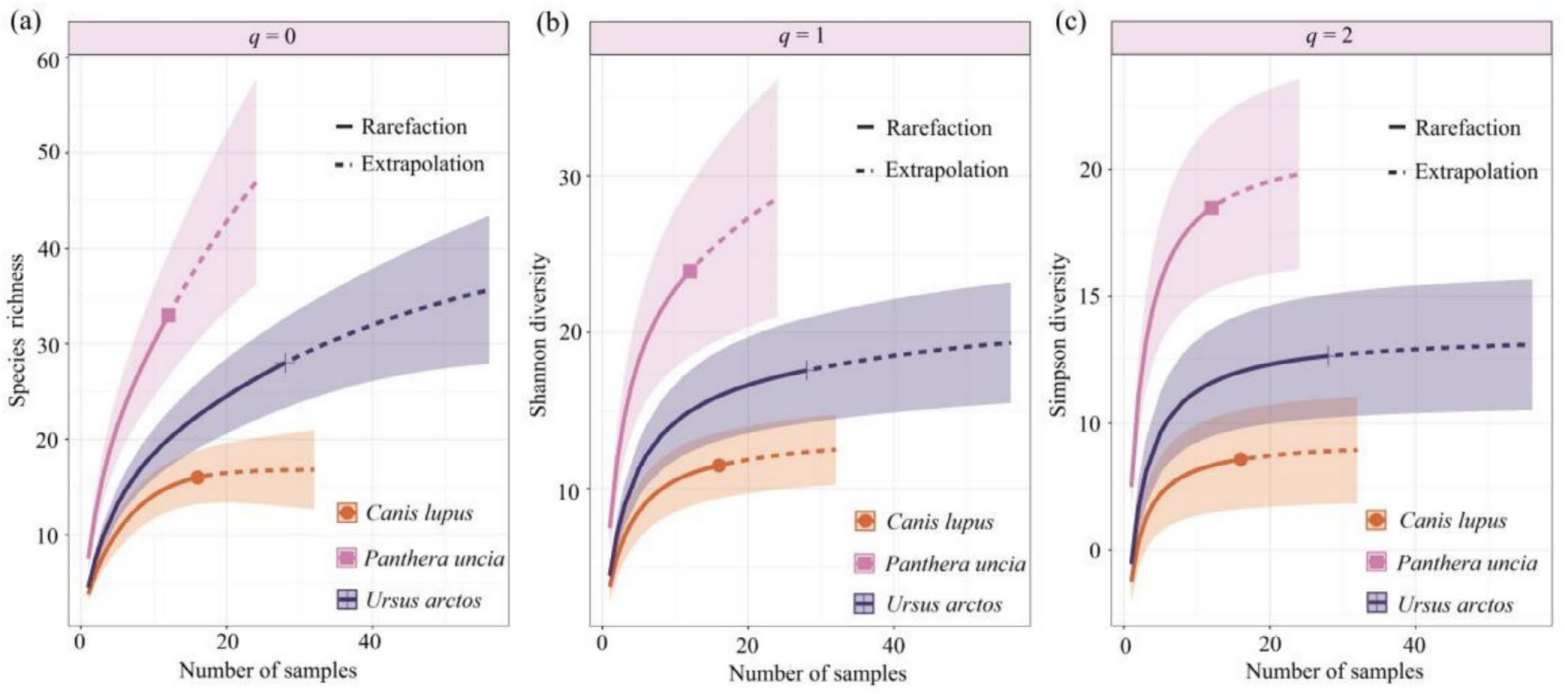
Hill number diversity rarefaction (solid lines) and extrapolation (dashed lines) curves for the dietary composition derived from fecal samples of three large carnivore species in the SR. Panel (a) displays the species richness rarefaction and extrapolation curves for dietary composition; panel (b) illustrates the Shannon diversity rarefaction and extrapolation curves; panel (c) presents the Simpson diversity rarefaction and extrapolation curves. The right endpoint of each rarefaction curve corresponds to the observed sample size, while the extrapolation curves extend up to twice this size. Shaded areas represent 95% confidence intervals.

By plotting the Hill number diversity rarefaction and extrapolation curves for species richness of the three large carnivore species at *q* = 0 (Figure 2a), *q* = 1 (Figure 2b), and *q* = 2 (Figure 2c), it was observed that the species richness curve for wolves reached a plateau after 15 fecal samples, indicating that the sample size for this species in the present study was sufficient. In contrast, additional fecal samples are required for snow leopards and brown bears to adequately capture the full extent of dietary species richness, particularly for the snow leopard. For brown bears, the rarefaction curves of both the Shannon diversity index and Simpson diversity index showed a tendency to approach saturation at the actual sample size; however, the indices continued to exhibit an increasing trend, suggesting that additional sampling may still contribute to the detection of new dietary taxa. In the case of snow leopards, the species diversity indices continued to rise beyond the current sample size, indicating that further collection of fecal samples is necessary to more comprehensively characterize their dietary composition.

### 3.2 Dietary Composition

**FIGURE 3:**
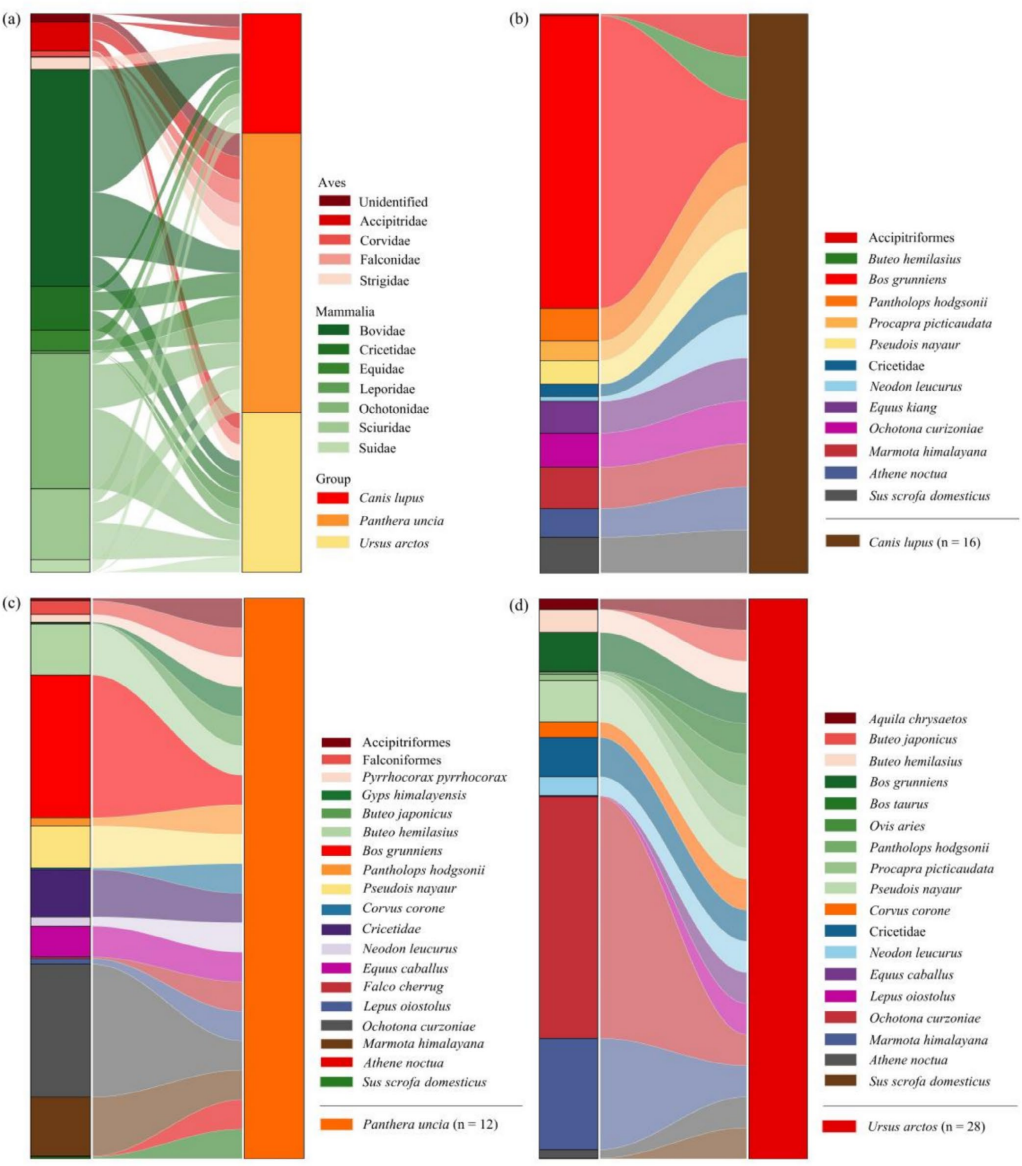
Dietary composition of three large carnivore species at the species level in the SR: (a) dietary composition at the family level; (b) *Canis lupus* ; (c) *Panthera uncia* ; (d) *Ursus arctos* . The color blocks on the left represent the relative read abundance (RRA), expressed as a percentage, of prey items identified at the family or species level, whereas those on the right indicate the sample sizes for each respective carnivore species, with block dimensions proportionally scaled to the number of samples. Numerical values in parentheses denote the number of fecal samples collected for each corresponding large carnivore species.

In the dietary composition of wolves (Figure 3a), the avian component comprised two families from the class Aves: Strigidae (*RRA* = 5.09%) and Accipitridae (*RRA* = 0.10%). The mammalian component included six families, among which Bovidae (*RRA* = 65.89%), Sciuridae (*RRA* = 7.39%), and Suidae (*RRA* = 6.42%) were the most prevalent. At the family level (Figure 3a), in addition to unidentified bird species, the dietary composition of snow leopards encompassed four families within the class Aves, with Accipitridae being the most dominant (*RRA* = 9.43%). Regarding the class Mammalia, seven families were identified, with Bovidae (*RRA* = 31.41%), Ochotonidae (*RRA* = 23.71%), and Sciuridae (*RRA* = 10.54%) representing the three most abundant. For brown bears, the dietary composition encompassed three avian families within the class Aves, with Accipitridae again representing the dominant family (*RRA* = 5.95%). Regarding the class Mammalia, seven families were represented, with Ochotonidae (RRA = 43.10%), Bovidae (RRA = 16.00%), and Cricetidae (*RRA* = 10.37%) being the top three in relative abundance.

**FIGURE 4:**
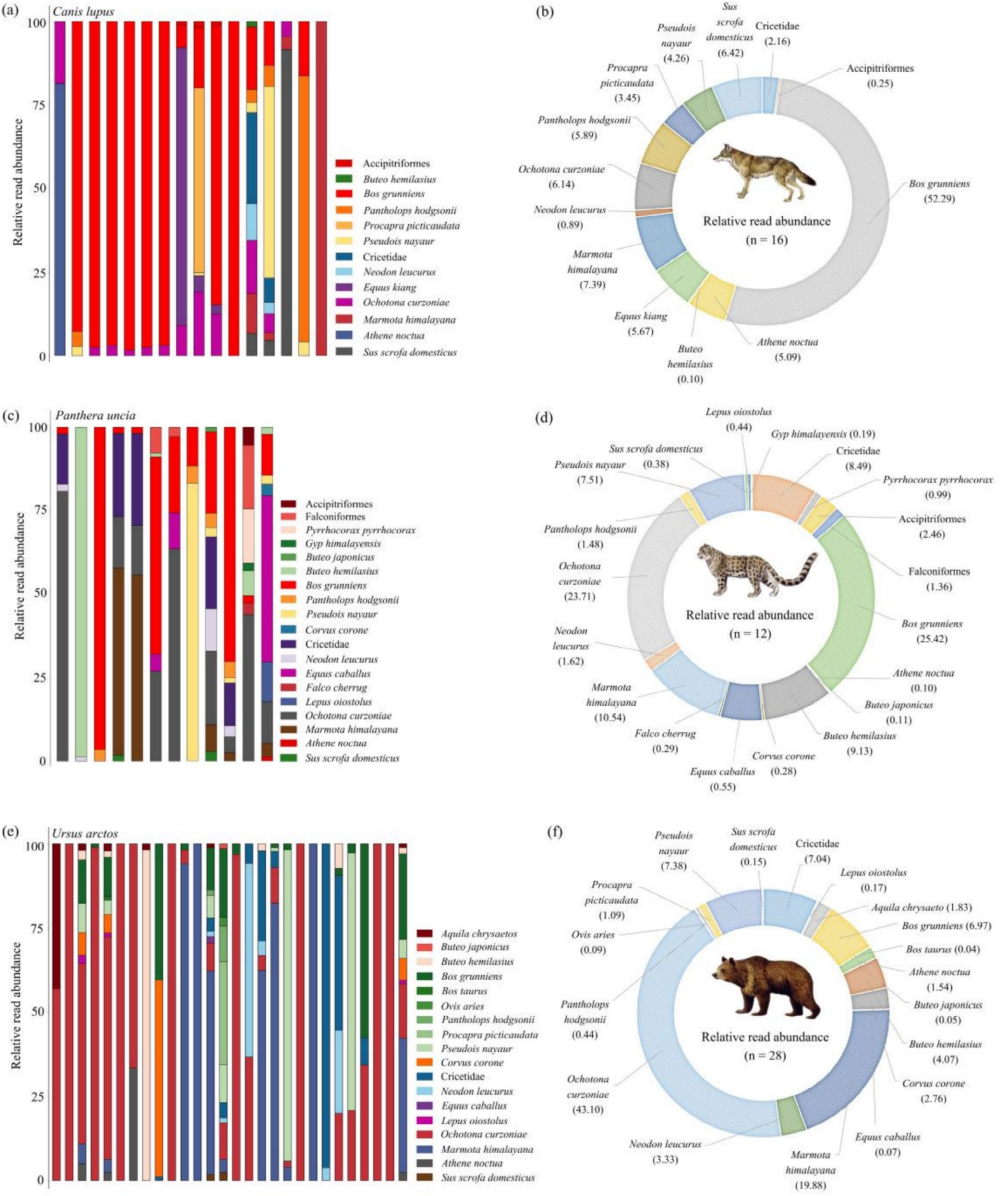
Dietary composition and relative read abundance of prey species for *Canis lupus* (panels a and b), *Panthera uncia* (panels c and d), and *Ursus arctos* (panels e and f) in the SR. The silhouettes of the three large carnivores are sourced from the ‘A Guide to the Mammals of China’ by Smith and Xie (2009). Each individual bar in the stacked bar charts on the left side (a, c, e) represents a single fecal sample.

At the species level (Figure 3b–d), excluding unidentified prey items, brown bears exhibited the highest number of prey species in their diet (*n* = 17), followed by snow leopards (*n* = 16) and wolves (*n* = 11). For wolves (Figures 3b and 4a,b), the dominant prey species based on percentage contribution were domestic yak ( *Bos grunniens* , *RRA* = 52.29%), Himalayan marmot ( *Marmota himalayana* , *RRA* = 7.39%), domestic pig ( *Sus scrofa domesticus* , *RRA* = 6.42%), plateau pika ( *Ochotona curzoniae* , *RRA* = 6.14%), and Tibetan antelope (*RRA* = 5.89%). For snow leopards (Figures 3c and 4c,d), the top five prey species in terms of relative abundance were domestic yak (*RRA* = 25.42%), plateau pika (*RRA* = 23.71%), Himalayan marmot (*RRA* = 10.54%), upland buzzard ( *Buteo hemilasius* , *RRA* = 9.13%), and bharal (*RRA* = 7.51%). In the case of brown bears (Figures 3d and 4e,f), among the 17 prey species detected, the top five species by *RRA* were plateau pika (*RRA* = 43.10%), Himalayan marmot (*RRA* = 19.88%), bharal (*RRA* = 7.38%), domestic yak (*RRA* = 6.97%), and upland buzzard (*RRA* = 4.07%).

### 3.3 Dietary Breadth and Overlap

Throughout the year, the brown bear (*B* = 0.38) exhibited the broadest trophic niche width, followed by the snow leopard (*B* = 0.30) and the wolf (*B* = 0.19). Pairwise calculations of niche overlap indices revealed considerable interspecific overlap on an annual scale: the trophic niches of snow leopards and wolves (*O*_*jk*_ = 0.76), as well as those of snow leopards and brown bears (*O*_*jk*_ = 0.79), exhibited high similarity. Compared to other species pairs, the trophic niche overlap between wolves and brown bears was substantially lower (*O*_*jk*_ = 0.32) yet remained statistically significant.

## 4 Discussion

Investigating the dietary composition and interspecific differences among large carnivore species not only enhances our understanding of their predation pressure, trophic niche differentiation, and intraspecific and interspecific competition dynamics, but also contributes to broader insights into their behavioral ecology and functional roles within ecosystems (Braczkowski et al. 2018). However, studying carnivores presents significant challenges due to their predominantly nocturnal, elusive, and wide‐ranging behavior, as well as their generally low population densities. Sign surveys and infrared camera trapping are currently the primary field techniques used to investigate their distribution, behavior, and abundance (Alexander et al. 2016; Wang et al. 2024). Nevertheless, these methods are limited in their ability to assess dietary composition and other ecological parameters. Recent technological advances have enabled the application of DNA metabarcoding, employing universal primers and second‐generation sequencing, to rapidly and effectively extract high–throughput species‐specific sequences from mixed fecal samples. This method has been widely adopted in dietary composition analysis and biodiversity studies, offering a more objective and accurate alternative to traditional morphological identification methods, which are prone to high error rates due to subjective interpretation of fecal characteristics (Lonsinger et al. 2015; Monterroso et al. 2013). In this study, fecal samples were collected from snow leopards, wolves, and brown bears in the SR during both warm and cold seasons. Utilizing DNA metabarcoding, we quantified dietary composition, seasonal variation, and interspecific dietary overlap among these four large carnivore species. The results provided insight into interspecific interference and competition intensity through the analysis of trophic niche partitioning. Additionally, this study demonstrated the efficiency and practicality of fecal DNA for rapid species surveys.

Carnivores typically exhibit selective predation, as demonstrated by regional and seasonal variations in the composition and proportion of prey in their diets. Such variations reflect, to a certain extent, the adaptability of predators to local environmental conditions (Ferretti et al. 2024; Selvan et al. 2013). Previous studies have shown that snow leopards inhabiting the Qinghai–Xizang Plateau primarily rely on large wild ungulates, with bharal constituting the dominant component of their diet (Lu et al. 2023). Additionally, research conducted in the Qilian Mountains reveals that snow leopard diets include domestic livestock and plateau pika in addition to bharal (Cong et al. 2024), indicating both similarities and differences compared to our findings. Our study found that the top three prey species by RRA in the snow leopard diet were domestic yak, plateau pika, and Himalayan marmot, with bharal ranked only fifth. This discrepancy may be attributed to regional variations in prey selection and dietary composition across snow leopard populations.

As group‐hunting predators, wolves are generally capable of hunting large prey, such as wild ungulates including kiang and wild yak (Lu et al. 2023; Shao et al. 2021). However, our dietary analysis revealed that domestic yaks constituted approximately half of the wolf diet in the SR (*RRA* = 52.29%), with the remainder primarily consisting of wild ungulates (*RRA* = 19.63%). Brown bears, as opportunistic omnivores, exhibit a highly diverse diet, predominantly consisting of plateau pikas and Himalayan marmots (Cong et al. 2024; Dai et al. 2021; Xu et al. 2009). This is consistent with the findings of our study, which confirmed that plateau pikas (*RRA* = 43.10%) and Himalayan marmots (*RRA* = 19.88%) constitute the primary prey items in the dietary composition of brown bears. However, previous studies have demonstrated that, in addition to animal prey, the diet of brown bears also includes plant matter, grains, and ghee, among other food sources (Dai et al. 2021). These components were not detected in the brown bear fecal samples analyzed in our study. A possible explanation is that the barcoding primers used in this research were specifically designed for vertebrates (Riaz et al. 2011), potentially leading to omissions in the dietary assessment of omnivore species. Therefore, to obtain a more comprehensive understanding of the dietary composition of brown bears, future studies should incorporate plant‐specific primers and combine molecular techniques with microscopic examination methods for more accurate dietary reconstruction. Due to sampling constraints, we were unable to completely avoid pseudo‐replication of fecal samples. To mitigate its influence, future studies should expand the spatial extent of sampling and refrain from collecting multiple morphologically similar samples within a limited area. However, to fully eliminate this confounding factor, individual identification of scats through genetic or other diagnostic methods is recommended, ensuring that only one sample per individual is included in experimental and statistical analyses. Furthermore, we aimed to avoid including fecal samples collected during the same period that originate from a single predation event. To minimize pseudo‐replication and ensure an accurate representation of dietary composition, we recommend sampling multiple individuals across distinct time periods.

Wolves and brown bears are widely distributed across Eurasia and North America. Research has shown that wolves in North America exhibit a strong year‐round dependence on ungulate prey. This pattern is reflected in regional dietary variations: in east‐central Minnesota, USA, the white‐tailed deer ( *Odocoileus virginianus* ) constitutes the primary prey, with the mountain hare ( *Lepus timidus* ) serving as a secondary food source (Fuller 1997); in the western Deccan Peninsula of India, the diet is dominated by blackbuck ( *Antilope cervicapra* ), followed by domestic sheep and goats (Kamlesh et al. 2011); in central Italy, wild boar ( *Sus scrofa* ) is the principal prey species (Pezzo et al. 2003). On the Qinghai–Xizang Plateau of China, wolves primarily prey on ungulates during the warm season but supplement their diet with rodents. However, during the relatively food–scarce cold season, predation on domestic yaks by wolves increases significantly (Shao et al. 2019; Lu et al. 2023). In general, prey encounter rate, vulnerability, and availability are the primary factors influencing wolf prey selection (Lisa and Rebecca 2003). Variation in these factors accounts for the significant dietary differences observed among wolf populations across different regions. Over the year, brown bears exhibit distinct temporal patterns in key activities such as mating, hyperphagia, and winter dormancy, representing characteristic ecological phases that may influence dietary composition (Pauly et al. 2025). Furthermore, the diet of the brown bear demonstrates high plasticity, varying considerably across geographic regions. In the Pyrenees of southwestern Europe, the brown bear's diet is predominantly plant–based, supplemented by arthropods, vertebrates, and fungi (Pauly et al. 2025). In North America, salmon constitute a critical food resource during their spawning migrations into freshwater systems (Mowat and Heard 2006). On the Qinghai–Xizang Plateau, rodents, particularly the plateau pika and Himalayan marmot, are the primary food source, with plants, grains, and livestock also contributing to the diet (Dai et al. 2021). The primary prey of snow leopards varies significantly across their distribution range, with markhor ( *Capra falconeri* ) predominating in Pakistan, Siberian ibex ( *Capra sibirica* ) in Mongolia and Kyrgyzstan, and bharal in China (Hacker et al. 2021). The distribution of the snow leopard largely overlaps with that of its wild ungulate prey. While diet composition is influenced by local food resource diversity, it is primarily dominated by mountain ungulates and livestock, with small mammals and birds serving as supplementary components (Wang et al. 2014; Shrestha et al. 2018).

Human–wildlife conflicts are widespread in the SR, primarily driven by livestock predation by large carnivores such as snow leopards, wolves, and brown bears (Dai et al. 2020; Li et al. 2013). Previous studies have indicated that more than half of large carnivore species exhibit substantial spatial overlap with grazing livestock (Xiao et al. 2022). Among them, species such as the snow leopards show the highest degree of habitat overlap, with approximately 80% of their range coinciding with areas used by grazing livestock (Li and Lu 2014). Compared to wild prey, livestock are more accessible and abundant. Consequently, when spatial use patterns between large carnivores and livestock highly overlap, it is likely to result in increased livestock predation (Dai et al. 2020; Ji et al. 2022). Over time, this situation may prompt herders to take retaliatory actions against individual animals responsible for attacking livestock. It is estimated that approximately 11 snow leopards die annually in the SR due to poaching and retaliatory killing (Li and Lu 2014). Moreover, the presence of animal traps and individuals with missing limbs further corroborates the occurrence of retaliatory hunting (Wang et al. 2024). The proportion of livestock in the dietary composition of the three large carnivore species is consistent with our previous Prediction 1. We found that domestic livestock constituted the largest proportion in the dietary composition of wolves (*RRA* = 52.29%), followed by snow leopards (*RRA* = 30.92%) and brown bears (*RRA* = 7.13%). This finding further underscores the prevalence of human–wildlife conflict in the SR. In particular, the finding that domestic livestock constituted the majority (> 50%) of the wolf diet supports previous research indicating that local livestock losses are primarily attributable to wolf predation (Dai et al. 2020; Yan et al. 2019). However, DNA metabarcoding is unable to determine the number of prey individuals, distinguish between predation and scavenging, or provide information on the age and size of prey. Furthermore, this method does not yield information on other demographic characteristics of prey items (Pompanon et al. 2012; Hacker et al. 2022). We speculate that, with the exception of the omnivorous brown bear, livestock consumed by wolves and snow leopards is primarily derived from active predation. This inference is consistent with the findings of previous studies based on microscopic fecal analysis: the proportion of livestock in the diet of snow leopards in the Qinghai region is relatively high (Li 2012), and contributions ranging from approximately 38% to 78% in the wolf's diet (Liu and Jiang 2003). In the SR, domestic livestock populations substantially exceed those of wild ungulates. Their high abundance renders them a primary food resource for large carnivores such as wolves and snow leopards (Wang et al. 2024). Our findings confirmed the substantial reliance of these carnivores on free–ranging livestock. Nevertheless, future research is required to determine whether dietary livestock is acquired through active predation and/or scavenging and to quantify the relative contribution of each.

Over the course of our background investigation, we found that livestock in the SR primarily consist of domestic yaks, horses, and sheep, with no evidence of pig or yellow cattle farming in the region. Nevertheless, non–locally farmed species such as pigs and yellow cattle were detected in the diets of all three large carnivore species. A plausible explanation was that herders discarded leftover bones in the wild during grazing activities, which were commonly scavenged by carnivores. Additionally, the presence of non–local prey items in brown bear diets might be attributed to their foraging behavior on kitchen refuse.

The degree of dietary niche overlap serves as an indicator of the potential intensity of competitive interactions between species (Du Preez et al. 2017). Furthermore, size disparity among carnivore species significantly influences the extent of such dietary niche overlap (Lanszki et al. 2019). The results of this study indicated a high degree of dietary overlap between snow leopards and wolves (*O*_*jk*_ = 0.76), as well as between snow leopards and brown bears (*O*_*jk*_ = 0.79). However, the similarity between their diets is relatively low, suggesting that while there is some overlap, these species engage in resource partitioning through differential prey selection, thereby reducing direct competition. A possible explanation for the high dietary overlap yet low similarity among species is that the former represents a quantitative index derived from numerical calculations, whereas the latter is based on qualitative differences in species composition. This adaptive strategy enables them to mitigate excessive competition for food resources in sympatric occurrences by selecting different prey species and exploiting varying levels of prey availability. In contrast, the lower dietary overlap observed between wolves and brown bears (*O*_*jk*_ = 0.32) indicated that trophic niche differentiation facilitates their coexistence within the SR. The trophic niche overlap indices for species pairs reveal that only the high dietary niche overlap between brown bears and snow leopards contradicts our previously proposed Prediction 2.

The trophic niche breadth of an animal characterizes its trophic position and dietary interactions within the ecosystem (Crozier 1985). This study found that among the three large carnivores, the brown bear, an omnivorous opportunistic forager, exhibited the widest dietary niche breadth, whereas the wolf, which primarily relies on a pack‐hunting strategy, displayed the narrowest. This pattern arises because brown bears exploit a wide range of available food resources in their environment, resulting in a broader niche. In contrast, wolves predominantly consume large ungulates, leading to a relatively narrower dietary niche. These differences enable complementary utilization of food resources among the large carnivores and thereby reduce interspecific competition. According to optimal foraging theory, dietary niche breadth tends to narrow when food resources are abundant and expand under resource scarcity, as a means of maintaining energy intake rates (Huisman and Weissing 1999; Steinmetz et al. 2021). Due to limitations in sample size, we did not analyze whether seasonal variations in dietary niche breadth among the three large carnivores align with the predictions of optimal foraging theory. Therefore, future research should involve the collection of fecal samples across multiple seasons to enable comparisons of seasonal shifts in dietary composition and the degree of dietary differentiation among species.

This study employed DNA metabarcoding to preliminarily elucidate the vertebrate dietary composition and niche overlap among snow leopards, wolves, and brown bears in the SR. However, due to sampling constraints, the relatively limited number of fecal samples may have affected the accuracy of the results. Additionally, in some collected samples, DNA extraction and subsequent PCR amplification for sequencing were hindered by poor sample quality or the presence of PCR inhibitors. Therefore, future studies should prioritize optimizing DNA extraction protocols and increasing the collection of fresh fecal samples to improve the accuracy of dietary composition analysis and a better understanding of interspecific relationships among these three large carnivores.

In summary, the coexistence of snow leopards, wolves, and brown bears is facilitated through behavioral mechanisms involving prey selection and niche partitioning. Effective prey selection is feasible only when appropriate prey size classes are sufficiently available and not limiting (Karanth and Sunquist 1995). We infer that the adequate availability of wild prey species, such as kiang, bharal, Himalayan marmot, plateau pika, and mountain hare, enables these large carnivores to coexist within the SR of the Qinghai–Xizang Plateau.

## 5 Conclusions

Understanding the dietary composition and differences among sympatric large carnivores is crucial for gaining deeper insights into their interspecific interactions and resource partitioning. This knowledge is of critical importance for species conservation and the maintenance of ecological balance. In this study, by analyzing the species composition and relative proportions of vertebrate prey in the diets of three sympatric large carnivores in the SR, we found that the wolf, snow leopard, and brown bear differed significantly in prey species composition and exhibited considerable variation in the consumption of shared prey. Interspecific relationships suggested that wolves and brown bears achieved coexistence through dietary niche differentiation. Although the snow leopard exhibited high dietary niche overlap with both the wolf and the brown bear, interspecific competition might be mitigated by differential selection of prey resources and variation in consumption proportions. Moreover, the substantial contribution of livestock to the diets of wolves and snow leopards reflected a significant reliance on domestic animals, indicating an underlying human–wildlife conflict in the SR. This issue warrants urgent attention and the implementation of effective mitigation measures.

## Author Contributions

**Dong Wang:** conceptualization (equal), data curation (lead), formal analysis (lead), investigation (equal), methodology (equal), resources (equal), software (lead), visualization (equal), writing – original draft (lead), writing – review and editing (lead). **Quanbang Li:** formal analysis (equal), investigation (equal), methodology (equal), resources (equal). **Xinming Lian:** conceptualization (lead), data curation (lead), formal analysis (lead), funding acquisition (lead), investigation (equal), methodology (lead), project administration (equal), software (lead), supervision (lead), visualization (lead), writing – original draft (equal), writing – review and editing (lead).

## Funding

This work was supported by the Regional Innovation and Development Joint Funds of the National Natural Science Foundation of China, U24A20361. National Key Research and Development Program of China, 2023YFF1305000.

## Conflicts of Interest

The authors declare no conflicts of interest.

## Supporting information


**Data S1:** ece373037‐sup‐0001‐DataS1.xlsx.

## Data Availability

All the required data are uploaded as Supporting Information.
